# The Treatment of Fractures

**Published:** 1901-10

**Authors:** 


The Treatment of Fractures. By W. L. Estes, A. M., M. D., Director and Physician and Surgeon-in-Chief of St. Luke's Hospital, South Bethlehem, Pa. Pp. 220; price $2.00. Published by the International Journal of Surgery Co., 100 William Street, New York.

This book is based mainly upon the experience of the author. This fact is worth stating, for in the matter of book making it is not the most common thing to see one's own experience given much prominence. Authors are sometimes modest men, and besides, some authors do not seem to have had much experience.

This little book covers the subject in a practical way, and has many cuts to illustrate the various fractures and their treatment.

